# New Drugs and Preparations

**Published:** 1894-03-10

**Authors:** 


### NEW DRUGS AND PREPARATIONS.

[We shall be glad to receive, at our Office, 428, Strand, London, W.C., from the manufacturers, specimens of all new preparations and drugs which may be brought out from time to time.]

### SOME EFFECTS OF ANTIPYRIN, ACETANILIDE, AND PHENACETIN.

Paterson[1] gives an account of a collective investigation into the untoward effects of these drugs carried on by twenty-five general practitioners in South Wales. Antipyrin was usually given to reduce the temperature or to relieve pain. For the first purpose, doses of 10 to 20 grains were used with little ill-effect. To relieve pain it is very largely prescribed, especially for sick headache and neuralgia. For this purpose, 5 to 15 grains were usually given. Of the twenty-five reports, seventeen note untoward symptoms, varying from unpleasant sweating to severe collapse. In one case 10 grains, and in another 20 grains produced dangerous collapse. Antipyrin seems especially liable to produce collapse in pneumonia, apparently from cardiac failure. Two reports mention that a rash appeared. In all the recorded cases the rash is a variety of erythema. Acetanilide is not nearly so much used, because it more frequently produces symptoms of poisoning. In pneumonia it is especially unsafe, because it so often leads to severe collapse. Phenacetin is certainly the least dangerous, and on the whole the best to use to relieve pain, but its antipyretic power is feeble. Cerna and Carter[2] find that in small doses antipyrin raises the blood pressure and increases the pulse rate; in large doses it diminishes the pressure and slows the pulse, in both cases acting on the heart. They therefore agree that it is not a desirable drug. They also believe that it reduces the heat production, and also the heat dissipation. Phenacetin they find is more stimulant to the heart than antipyrin in the first stage of its action, and less depressant in the second. It reduces the temperature in the same way as antipyrin, and is, on the whole, a much superior drug. They maintain that neither will reduce the normal temperature. An editorial article,[3] however, points out that although these drugs do not lower the normal temperature, still they diminish the production of heat, and therefore, perhaps, people who take them habitually to relieve pain are especially liable to catch cold. Pattison[4] reports three cases, two of them being children, who were severely collapsed, one after the use of antipyrin, one after acetanilide, and one after phenacetin. He also mentions the case of a Hindu, who recovered after taking 120 grains of antipyrin. Cappelletti[5] mentions the case of a girl who, without the consent of her doctor, gradually increased her dose of antipyrin till she was taking 120 grains a day. This brought on a condition in which she lost appetite, took no interest in domestic affairs, was very liable to severe convulsions, had bad headaches, and a buzzing in her ears. Nevertheless, she became greatly excited if the usual dose was withheld. She was put in an asylum, the attempt to stop the antipyrin suddenly produced dangerous symptoms of collapse, and the only way she could be cured was to reduce it gradually and give her bromide of potassium and caffeine. Horbaczewski[6] finds that both antipyrin and acetanilide diminish the amount of uric acid eliminated and increase the number of white blood corpuscles, and, on the whole, thinks there are many points of antagonism between the action of these drugs and quinine. Gleason[7] strongly recommends the local use of solutions of 1 in 20 of antipyrin for chronic and acute inflammations of the pharynx, nose, and larynx.

[1] Practitioner, Oct., 1893. [2] Therapeutic Gazette, March, 1893. [3] Therapeutic Gazette, 1893. [4] Indian Medical Record, June, 1892. [5] Ri. Sper. di Freniatria e di Med. Reg., March 31, 1893. [6] Revue de Thérapeutique Générale et Thermale, Sep. 20, 1892. [7] New York Medical Journal, Oct. 29, 1892.

